# Difference in performance between 3D and 4D CBCT for lung imaging: a dose and image quality analysis

**DOI:** 10.1120/jacmp.v17i6.6459

**Published:** 2016-11-08

**Authors:** Sheeba Thengumpallil, Kathleen Smith, Pascal Monnin, Jean Bourhis, François Bochud, Raphaël Moeckli

**Affiliations:** ^1^ Institute of Radiation Physics Lausanne University Hospital Lausanne Switzerland; ^2^ Swiss Federal Institutes of Technology Lausanne (EPFL) Lausanne Switzerland; ^3^ Radio‐Oncology Department Lausanne University Hospital Lausanne Switzerland

**Keywords:** 3D CBCT, 4D CBCT, breathing variations, free‐breathing radiation therapy

## Abstract

The study was to describe and to compare the performance of 3D and 4D CBCT imaging modalities by measuring and analyzing the delivered dose and the image quality. The 3D (Chest) and 4D (Symmetry) CBCT Elekta XVI lung IGRT protocols were analyzed. Dose profiles were measured with TLDs inside a dedicated phantom. The dosimetric indicator cone‐beam dose index (CBDI) was evaluated. The image quality analysis was performed by assessing the contrast transfer function (CTF), the noise power spectrum (NPS) and the noise‐equivalent quanta (NEQ). Artifacts were also evaluated by simulating irregular breathing variations. The two imaging modalities showed different dose distributions within the phantom. At the center, the 3D CBCT delivered twice the dose of the 4D CBCT. The CTF was strongly reduced by motion compared to static conditions, resulting in a CTF reduction of 85% for the 3D CBCT and 65% for the 4D CBCT. The amplitude of the NPS was two times higher for the 4D CBCT than for the 3D CBCT. In the presence of motion, the NEQ of the 4D CBCT was 50% higher than the 3D CBCT. In the presence of breathing irregularities, the 4D CBCT protocol was mainly affected by view‐aliasing artifacts, which were typically cone‐beam artifacts, while the 3D CBCT protocol was mainly affected by duplication artifacts. The results showed that the 4D CBCT ensures a reasonable dose and better image quality when moving targets are involved compared to 3D CBCT. Therefore, 4D CBCT is a reliable imaging modality for lung free‐breathing radiation therapy.

PACS number(s): 87.57.C‐, 87.57.uq, 87.53.Ly

## I. INTRODUCTION

4D image‐guided radiotherapy (IGRT) plays an important role in ensuring the precise positioning of the tumor within the breathing cycle, as well as visualizing the tumor trajectory. This makes it possible to define small margins, thereby sparing healthy tissues.^(1)^

One proposed 4D IGRT approach is respiratory‐correlated cone‐beam CT (4D CBCT).^(2)^ The 4D CBCT dataset provides several features not available in 3D CBCT such as the 3D trajectory determination of the moving target and a specific algorithm for reducing motion artifacts. These features allow us to define patient's specific treatment margins. In 4D CBCT acquisitions, similarly to respiratory‐correlated CT (4D CT), the images are retrospectively binned according to the respiratory phase (usually 10 phases are reconstructed). This reduces respiratory motion artifacts and yields a 3D image at different tumor positions within the respiratory cycle. The tumor position can be compared to the planning phase (e.g., midventilation CT phase).^(3)^ The tumor motion verification and the correction of the tumor baseline shift based on a registration between the planning 4D CT and 4D CBCT is made prior to delivery, thus improving the precision of dose delivery.

Nevertheless, breathing irregularities may substantially reduce the image quality and consequently compromise the dose delivery,^2^, ^4^ especially in the case of lung free‐breathing radiation therapy (FBRT). Image quality is also strongly influenced by image acquisition parameters, such as dose per projection and the number of projections. Indeed, the 3D CBCT and the 4D CBCT are characterized by different image acquisition parameters, affecting dose and image quality in a different manner. This is explained by a slower gantry rotation necessary to ensure adequate angular sampling^(5)^ and more projections are needed to reconstruct more than one CBCT in different breathing phases. Therefore, many respiratory cycles occur during the 4D CBCT acquisition.^(2)^ The aim of this study was to evaluate the differences between the 3D and 4D CBCT Elekta XVI protocols^(6)^ in terms of dose and image quality, and to investigate their implications on the IGRT accuracy of lung FBRT.

## II. MATERIALS AND METHODS

### A. XVI CBCT imaging system and lung IGRT protocols

The CBCT acquisitions were performed with the Elekta Synergy linac imaging system (XVI System, Elekta AB, Stockholm, Sweden) mounted on the gantry perpendicular to the treatment beam. The XVI system is composed of an X‐ray tube with 5.25 mm Al inherent filtration and an amorphous silicon flat‐panel detector.^(7)^

Available XVI lung IGRT protocols are “Chest” for 3D CBCT and “Symmetry” for 4D CBCT. The Chest protocol performs a 360° rotation in 2 min (660 projections) with 40 mA tube current compared to 200° in 4 min (1320 projections) and 20 mA tube current for the Symmetry protocol. Indeed, the 4D CBCT protocol is built in such a way that an increase in the number of projections is compensated by an equivalent decrease of the X‐ray tube current. The Chest protocol uses a M20 kV collimator, in which the imager is laterally shifted by 10 cm, resulting in an asymmetric beam.^(6)^ As can be inferred from its name, the Symmetry protocol results with symmetric beam, with the imager aligned at the center of the X‐ray beam. The Chest protocol uses an aluminum bow‐tie filter of 2 mm thick at its center and 30 mm at the borders. This leads to a differential filtration of the X‐ray beam across the profile, whereas the Symmetry protocol does not use any additional filtration. The raw projections acquired with the Chest protocol are processed with the Feldkamp algorithm^(8)^ to reconstruct the CBCT slices. In the Symmetry protocol, the respiratory signal is directly extracted with the Amsterdam Shroud method,^(2)^ where each cone‐beam projection is a snapshot representing a given respiratory phase. The corresponding projections are sorted into several phase bins, which are then fed into the basic Feldkamp algorithm to generate a 4D CBCT dataset. In order to give a full description of the 3D (Chest) and 4D CBCT (Symmetry) protocols available in clinic, we kept the same acquisition parameters as proposed by the manufacturer for the analysis.

### B. Phantom data study: dose analysis

The usual computed tomography dose index (CTDI)^(9)^ is not valid when considering CBCT geometry, and it was therefore replaced by the cone‐beam dose index (CBDI) defined as:^7^, ^9^, ^10^

$$CBDI=\frac{1}{L}\int_{-Y/2}^{Y/2}D(y)dy$$

where *L* is the collimation width and *Y* is the length of dose integration. For usual CTDI measurements, the International Electrotechnical Commission (IEC) recommends using Y=100 mm.

However, this length is too small to cover the wide diffusion tails of CBCT and some authors^(11)^ suggested taking a larger dose integration length (approximately 400 mm). Accordingly, we performed measurements with Y=100 mm and 450 mm in order to quantify the difference between two extreme conditions. We also calculated the weighted CBDI defined as:

$${\text{CBDI}}_{w}=\frac{1}{3}{\text{CBDI}}_{center}+\frac{2}{3}{\text{CBDI}}_{periph}$$

where ${\text{CBDI}}_{\text{center}}$ is the CBDI measured in the central axis of the phantom and ${\text{CBDI}}_{\text{periph}}$ is the average of the CBDI measured at the four peripheral positions. *The* ${\text{CBDI}}_{w}$ is an indicator representative of the volumetric average dose within the phantom similar to the CTDI, as described in the report of American Association of Physicist in Medicine (AAPM) Task Group 23.^(12)^

For the CBDI measurements, we used a custom‐made phantom designed for this specific purpose. It is made of polymethylmethacrylate (PMMA) and is representative of a patient's trunk. It has a diameter of 320 mm and is 450 mm long. The original phantom length was 150 mm, but it was increased to cover the wide diffusion tails of the dose profile associated with the larger beam width of CBCT. The phantom contains five holes in the longitudinal direction: one at its center and four at 1 cm from the surface, at 90° from each other. These holes are filled with removable cylindrical inserts made of PMMA and drilled each 5 mm interval to allow lithium fluoride (LiF) thermoluminescent dosimeters (TLDs) to be placed for dose measurements.

In our measurements, the TLDs were placed every 15 mm along each insert of the phantom. The dose profiles were measured along the whole 450 mm phantom length. Given the sensitivity of the TLDs, each measurement consisted of two consecutive irradiations in order to deliver enough dose in the diffusion tails.

### C. Image quality analysis

Image quality analysis was performed with different phantoms aimed for specific measurements. To simulate breathing motion, the phantoms were mounted on the CIRS dynamic platform (CIRS, Norfolk, VA) producing a periodic movement described by the cosine power to 4, because it describes a typical asymmetric respiratory cycle in free breathing motion.^(13)^ The dynamic platform was submitted to a movement of 2 cm amplitude in the longitudinal direction and 0.25 Hz frequency. The alignment of the phantoms was checked using the treatment room lasers.

The point spread function (PSF_y_) in the longitudinal direction (y) was measured with CIRS AAPM CT performance phantom (CIRS) using the slice sensitivity profile (SSP) insert part, which contains three aluminum ramps aligned vertically and which rise at 23° angle from the base to the top of the phantom. The contrast transfer function (CTF_y_) is the modulus of the Fourier transform of the PSF_y_ along the motion direction and it was measured in the y direction from the slice where the signal of the ramp was maximum in the z direction. The CTF_y_ was used to assess the longitudinal resolution and contrast losses due to partial volume effects generated by the object motion.

The noise power spectrum (NPS_xz_) and the modulation transfer function (MTF_xz_) were measured in the transverse plane (xz‐plane), using a custom‐made cylindrical phantom of 25 cm diameter filled with water containing a cylindrical insert of Teflon (diameter 10 cm) at its center.^(14)^ The NPS_xz_ was calculated according to the IEC 62220‐1‐1 report^(15)^ within a 128×128 ROI chosen at the center of a homogeneous water region inside the phantom and averaged over 20 slices. The MTF_xz_ was determined from an edge analysis of the cylindrical Teflon insert as described elsewhere.^(14)^ The MTF_xz_ and NPS_xz_ were used to compute the 2D noise‐equivalent quanta (NEQ_xz_) in the transversal slices, which stands for signal‐to‐noise ratio in the spatial frequency domain, as described in the International Radiation Commission Unit (ICRU) Report 54^(16)^ and in Eq. (3):

$$NEQ_{xz}(f)=\frac{S^{2}\cdot MTF_{xz}^{2}(f)}{NPS_{xz}(f)}$$

where $f=\sqrt{f_{x}^{2}+f_{z}^{2}}$ is the radial frequency in the transverse plane and *S* is the peak signal value obtained from the PSF_y_ and reports signal losses due to partial volume effects generated by the motion in the longitudinal direction.

The image quality analysis of the Symmetry protocol was performed on the average image of 10 phases (average pixel values of each phase) and also on a single phase of the breathing cycle. The single phase was chosen to be the 60% phase representing the breathing cycle portion in which the tumor spends most of its time (midventilation phase). In addition, each breathing phase was subject to the same acquisition parameters and, therefore, we did not expect any change in the contrast and in the noise evaluation if we had chosen another phase.

### D. CBCT motion artifacts evaluation

The CIRS Dynamic Thorax Phantom (CIRS) was used to acquire CBCT images in the presence of irregular breathing variations. A 3 cm diameter sphere was moved inside the phantom along the longitudinal axis with an equation of motion of the sphere center defined by:

$$z(t)=A{\text{cos}}^{4}(2\pi tf)$$

where *t* is the time, *A* is the motion amplitude of the sphere, and *f* is the respiratory cycle frequency.

Three kinds of irregular breathing variations were randomly generated in the longitudinal direction. They were generated for: (i) amplitude variation (0.5–1.3 cm) and fixed frequency of 0.25 Hz, (ii) frequency variation (0.18–0.32 Hz) and fixed amplitude of 1 cm, and (iii) combined amplitude and frequency breathing variations (0.5–1.1 cm) amplitude and (0.23–0.26 Hz) frequency variations. These irregular breathing variations were representative of typical patient respiratory cycles.^(17)^

We also performed static acquisitions. Firstly, the images acquired with motion were compared to the images acquired without motion to qualitatively determine motion artifacts and CBCT artifacts. Secondly, the 3D and the 4D CBCT motion artifacts were quantitatively assessed by comparing the measured internal tumor volume (ITV) in the presence of motion artifacts with the theoretical ITV given by:^(18)^

$$ITV=43\pi r^{3}+L\pi r^{2}$$

where *L* is the maximal displacement of the sphere (tumor) and *r* is the radius of the sphere (15 mm).

The theoretical values were calculated for maximum motion amplitude and average motion amplitude. The measured ITV in the presence of motion artifacts was obtained for the Chest protocol by taking the 3D CBCT image, which directly shows the visible ITV^(4)^ and for the Symmetry protocol by taking the 4D CBCT images at 0% and 50%, corresponding to the maximum inspiration and maximum expiration phases, respectively.

## III. RESULTS

### A. Dose profiles and CBDI

**Figure 1:**
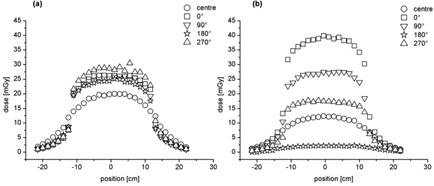
Chest (a) and Symmetry (b) dose profiles at central (round marker) and at gantry angles of 0° (square marker), 90° (down triangle marker), 180° (star marker), and 270° (up triangle marker) as a function of the TLDs position inside the phantom.

The dose within the slices was much more homogeneous for the Chest protocol (Fig. 1(a)) than the Symmetry protocol (Fig. 1(b)). This is due to the fact that the tube rotates only by 200° around the phantom for the Symmetry protocol compared to 360° for the Chest protocol. Also, for the Chest protocol, the highest peripheral dose was observed at 270° gantry angle (Fig. 1(a)), because the kV beam irradiation starts and ends around this angle and more time is spent there in accordance with the acquisition settings. This is not the case for the Symmetry protocol, for which the highest dose was observed at 0° gantry angle (Fig. 1(b)) because the rotation is only 200°.

Compared to the Symmetry protocol, the CBDI_w_ of the Chest protocol is more than 36% higher for an integration length of 100 mm and more than 32% over an integration length of 450 mm (Table 1). For both protocols, a slightly higher CBDI_w_ is found for the 100 mm dose integration length compared to the 450 mm dose integration length. However, it is not significant.

**Table 1:** CBDI_100_ and the CBDI_450_, in the center and on the periphery and CBDI with the total error $\sigma =\sigma_{\text{stat}}+\sigma_{\text{syst}}$ the sum of the statistical and systematic errors

	*Center*	*0°*	*90°*	*180°*	*270°*	${\text{CBDI}}_{w}$
*CBDI*_*100*_ *(mGy)*						
Chest	−19.7±1.9	26.2±2.1	25.4±2.4	24.4±2.3	28.7±2.9	25.2±2.2
Symmetry	12.0±1.2	39.0±3.4	27.1±2.3	2.2±0.3	17.4±1.6	18.5±1.6
*CBDI*_*450*_ *(mGy)*						
Chest	20.4±1.5	24.7±1.8	23.9±1.7	23.3±1.7	27.0±2.0	23.3±1.7
Symmetry	12.5±0.9	36.3±2.6	25.4±1.9	2.4±0.2	16.6±1.2	17.6±1.3

### B. Image quality: contrast transfer function (CTF), noise power spectrum (NPS), and noise‐equivalent quanta (NEQ)

**Figure 2:**
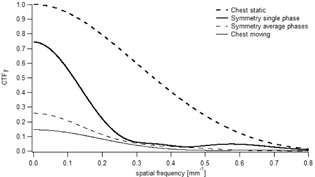
Normalized CTF_y_ along motion direction as function of the spatial frequency. The thick dashed line corresponds to the CTF_y_ of the Chest protocol acquired in static conditions, the thin line in moving conditions. The thick line represents the CTF_y_ of the Symmetry protocol evaluated in a single phase and the thin dashed line in the average phases.

The CTF_y_ in the presence of target movement is reduced (Fig. 2), leading to a significant lower CTF_y_ for the Chest compared to Symmetry protocol. In particular, by taking the Chest protocol acquired in static conditions as the reference, we find that the contrast at zero frequency is decreased by 25% for the Symmetry protocol evaluated in a single phase, by 65% for the Symmetry protocol evaluated in average phases, and by 85% for the Chest protocol acquired with motion.

**Figure 3:**
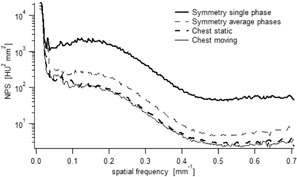
NPS for Chest and Symmetry protocols. The thick dashed line corresponds to the CTF_y_ of the Chest protocol acquired in static conditions, the thin line in moving conditions. The thick line represents the CTF_y_ of the Symmetry protocol evaluated in a single phase and the thin dashed line in the average phases.

The NPS show a similar frequency distribution among Chest and Symmetry protocols, and with or without movement (Fig. 3). However, the noise amplitude is the highest for the single phase evaluation of the Symmetry protocol. As expected, the noise amplitude of the Chest protocol is similar with or without movement.

**Figure 4:**
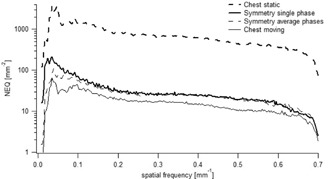
NEQ for Chest and Symmetry protocols. The thick dashed line corresponds to the CTF_y_ of the Chest protocol acquired in static conditions, the thin line in moving conditions. The thick line represents the CTF_y_ of the Symmetry protocol evaluated in a single phase and the thin dashed line in the average phases.

Similarly to the CTF_y_, the NEQ_xz_ is reduced when there is a movement (Fig. 4). For example, the NEQ_xz_ of the Chest protocol is about 50% lower than the Symmetry protocol evaluated in a single phase. Conversely, no significant difference is observed between the Symmetry protocol evaluated in a single phase and in the average phases.

### C. Image quality analysis: artifacts and internal tumor volume (ITV)

**Figure 5:**
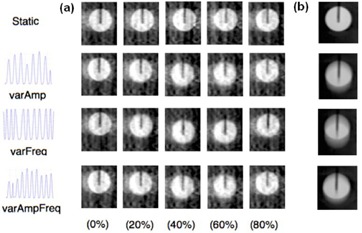
Examples of coronal images of the sphere for both static and moving acquisitions at different breathing phases obtained for the Symmetry protocol (a) and the Chest protocol (b). Each line corresponds to a different breathing variation.

In the presence of motion, view‐aliasing artifacts are visible in each breathing phase for the Symmetry protocol, whereas duplication artifacts are observed for the Chest protocol (Fig. 5).

Similar ITV volumes were measured between the Chest and the Symmetry protocols (Table 2); however, these volumes were smaller compared to the theoretical ones, for both maximum and average motion amplitude. Indeed, in the case of the amplitude breathing variation with maximum motion amplitude we found for both protocols an underestimation between 2% and 8% of the measured ITV compared to the theoretical value.

**Table 2:** Theoretical (Th) ITVs for maximum and average motion amplitude and measured ITVs for the Chest and the Symmetry protocols for the three kinds of breathing variations. The error on the measured ITV is the systematic error due to delineation

*Breathing Variation*	${\text{Th}}_{\text{max}}$ *(cc)*	${\text{Th}}_{\text{av}}$ *(cc)*	*Chest (cc)*	*Symmetry (cc)*
Amplitude	23.3	21.5	18.2±2.1	18.6±2.2
Frequency	21.3	21.3	20.3±2.8	20.2±2.8
Amplitude & Frequency	21.5	19.9	17.2±1.7	17.8±1.9

## IV. DISCUSSION

This study evaluated the performances of 3D and 4D CBCT Elekta XVI imaging protocols in terms of dose and image quality and their application on the IGRT accuracy of lung FBRT. In order to give a full description of the 3D (Chest) and 4D CBCT (Symmetry) protocols available in clinic, we kept the manufacturer's proposed acquisition parameters.

Compared to the Symmetry protocol, the dose profiles of the Chest protocol showed a more homogeneous dose distribution in all positions, due to the 360° gantry rotation compared to the 200° of the Symmetry protocol. We found cone beam dose index (CBDI) measurements in agreement with the findings of Amer et al.:^(6)^ the weighted CBDI_100_ was slightly higher, albeit not statistically significant, than the CBDI_450_. This allowed us to restrict measurements to the 100 mm central part of the image field of view (FOV) as they are representative of the average dose. In other words, a measurement across the 100 mm central part of the FOV gives a conservative estimate of the CBDI.

The CBDI_w_ for the Symmetry protocol was lower than that for the Chest protocol of 26% (see Table 1). Note that under equivalent arc rotation and acquisition parameters, the Symmetry protocol would have led to a higher nominal dose. Indeed, Lu et al.^(5)^ reported an increase of patient dose up to a factor of four compared to the 3D CBCT for a 5 sec breathing cycle period and the same acquisition parameters. To counteract this effect, the manufacturer reduced the X‐ray tube current and the gantry rotation to 200° in order to reduce dose and irradiation time, but at the cost of a decrease of image quality. Despite the only 200° arc rotation, the gantry speed is slowed down to get enough respiratory cycles to reconstruct the 4D CBCT scan. Thus, it causes a doubling of the irradiation time, 4 min compared to 2 min for a 3D CBCT scan. As a consequence, the increase of the irradiation time makes an increase in the risk of patient discomfort and position inaccuracy. Optimization of the protocols would reduce that risk; decreasing the image quality to a level guaranteeing an exploitable image by reducing the number of projections to a sufficient level would lead to a reduction of the irradiation time. For example, in our department we decided for lung FBRT to perform first a 4D CBCT of 4 min for patient setup and target match and after the treatment a 4D CBCT of 2 min, obtained by reducing half the number of projections, just for intrafraction motion assessment.

Motion may involve significant contrast loss due to partial volume effects and strong artifacts. Within this context, the common metric of contrast‐to‐noise ratio (CNR) may not be suitable, in the sense that a high CNR output may not indicate a better image quality.^(19)^ To quantitatively assess the loss of image quality due to motion for the two imaging protocols, we chose to use CTF and NEQ metrics, eliminating any dependency of contrast measurement on the sample size and shape. We found a severe loss of contrast when the motion was applied. The decreased contrast was lower for the Symmetry protocol compared to the Chest protocol.

Conversely, the noise is not affected by motion. However, the dose per projection, the number of projections and the algorithm used for slice reconstruction have an impact on noise. Hence, we found that the Symmetry protocol was more affected by noise than the Chest protocol. This can be explained by the fact that total delivered dose is lower for the Symmetry protocol compared to the Chest protocol. The NPS of CBCT slices scales with the inverse of the number of photons per projections, the NPS of the Symmetry protocol evaluated in average phases (1320 projections) and in a single phase (132 projections) were 2 and 15 times higher compared to the Chest protocol (660 projections), respectively. As expected, no difference in the Chest protocol was observed between static and moving acquisitions because motion does not contribute to the noise. By combining signal and noise transfer metrics, we could assess the NEQ that is giving the whole frequency information available to an observer looking at the image. However, in case of motion, a NEQ gain of 50% was observed for the Symmetry protocol compared to the Chest protocol. According to ICRU Report 41,^(20)^ the higher the NEQ, the higher the tumor visualization and the better image registration is between the planning CT and daily CBCT. Another important factor affecting image quality are the artifacts induced by respiratory motion. They may seriously compromise the precise registration between the planning CT and the daily CBCT and jeopardize the treatment outcome.^(4)^ We observed view‐aliasing artifacts when the images were acquired with the Symmetry protocol (phase‐by‐phase analysis). This was expected because the lower number of projections per phase led to undersampling (132 for the 4D CBCT single phase against 1320 projections of the 4D CBCT average phases).^(2,5,21–23)^ We observed no major difference between the static acquisition and the breathing variations for the 4D CBCT scan (Fig. 5). This is because the 4D reconstruction algorithm weights the projections by the gantry angle increment between the projections, in order to prevent artifacts.^(5)^ We found duplication artifacts for the Chest protocol due to the faster gantry rotation speed compared to the breathing cycle. This compromises the accuracy in terms of visualizing the tumor extent and image registration, but it also inhibits the direct use of CBCT for dose calculation.^(24)^

The artifacts also have a great impact on volume definition. We investigated the influence of the irregular breathing motions on tumor volume definition in images obtained by a CBCT system. We obtained similar measured and theoretical ITV volumes for both protocols. Nevertheless, we found a more important underestimation of the ITV volumes compared to the theoretical for both protocols, in the case of amplitude breathing variations. For the Chest protocol, this result is somehow expected, as the motion related partial volume artifacts will lead to reduced ITV. For the Symmetry protocol, the motion artifacts are greatly suppressed compared to the Chest; however, because of the poor image quality in each phase, the measurement of the ITV volume (union of the images at 0% and 50%, corresponding to the maximum inspiration and maximum expiration phases) becomes less accurate and in our case comparable to the Chest (Table 2). As observed by Vergalasova et al.,^(25)^ an underestimation of the ITV could lead to an improper alignment of the planning CT ITV with respect to the daily CBCT ITV. However, even if the results obtained from both protocols were similar, a precise tumor volume definition was harder to achieve with 3D CBCT because of the presence of larger artifacts, such as the duplication structure. This result was in agreement with the findings by Sweeney et al.,^(26)^ where they observed a larger variability in tumor visualization for the 3D compared to the 4D CBCT scan. Therefore, the 4D CBCT protocol is somewhat optimized in the context of a trade‐off between dose and image quality, though improvement can still be obtained.^(27)^ We found that the 4D CBCT ensures a reasonable dose and lower artifacts when moving targets are involved, compared to 3D CBCT. The above considerations support the use of 4D CBCT in the presence of motion and in the particular case for lung FBRT. It is a reliable lung imaging modality for accurate tumor localization and for safe margins reduction, further preventing the “geographical miss” of the tumor, especially in the presence of breathing irregularities. As a last comment we discuss the limitations of our study. A first limitation is that we only tested a 1D motion. However, the chosen direction (longitudinal) is the most relevant from a clinical point of view.^(28)^ Other limitations come from the fact that we used fixed amplitude and breathing frequency in the CTF analysis.

## V. CONCLUSIONS

The XVI Elekta 3D (Chest) and 4D CBCT (Symmetry) lung IGRT protocols have been evaluated in terms of dose and image quality. The two imaging modalities are efficiently optimized for the verification of patient setup prior to the treatment, as required in IGRT protocols. However, when dealing with respiratory motion, the 4D IGRT Symmetry protocol makes it possible to visualize the 3D tumor trajectory without additional respiratory monitoring and strongly reduces motion artifacts compared to 3D IGRT Chest protocol. Moreover, the 4D CBCT delivers an optimized dose to the patient and better image quality when moving targets are involved, compared to 3D CBCT. However, a drawback of a 4D CBCT is a longer irradiation time that may cause patient discomfort and position inaccuracy. This risk could be minimized in performing an optimization of the protocols. Therefore, having in mind the possible improvements that can be applied, we recommend the 4D IGRT Symmetry protocol based on 4D CBCT as a reliable imaging modality, whenever it is available, for lung FBRT treatments.

## COPYRIGHT

This work is licensed under a Creative Commons Attribution 3.0 Unported License.
